# Interaction between human osteosarcoma and mesenchymal stem cells via an interleukin-8 signaling loop in the tumor microenvironment

**DOI:** 10.1186/s12964-018-0225-2

**Published:** 2018-04-06

**Authors:** Masanori Kawano, Kazuhiro Tanaka, Ichiro Itonaga, Tatsuya Iwasaki, Hiroshi Tsumura

**Affiliations:** Department of Orthopaedic Surgery, Faculty of Medicine, Oita University, Oita, 879-5593 Japan

**Keywords:** Interleukin-8, Osteosarcoma, Mesenchymal stem cells, Tumor proliferation and metastasis

## Abstract

**Background:**

Osteosarcoma (OS) is the representative primary malignant bone tumor with the highest incidence. It is known that malignant phenotypes of OS, such as proliferation, invasion, and metastasis, are significantly influenced not only by characteristics of the tumor itself, but also by the surrounding microenvironment. In other words, OS is considered to utilize cells in the vicinity of the tumor by changing the characteristics of these cells. Direct intercellular contact is believed to be important for this phenomenon. In the present study, we hypothesized that an interaction mediated by a humoral factor, requiring no cellular contact, might play a significant role in the progression of OS.

**Methods:**

We developed a new co-culture model, using OS cells and mesenchymal stem cells (MSCs) without cellular contact, and found that both cell types expressed IL-8 at a high level, and FAK in OS cells was phosphorylated leading to an increase in the metastatic potential of the tumor in the co-culture condition.

**Results:**

It was revealed that OS cells formed a loop of signal cross-talk in which they released IL-8 as a paracrine factor, stimulating MSCs to express IL-8, and received IL-8 released by MSCs to accelerate IL-8 expression in OS cells. Administration of anti-IL-8 antibody resulted in the inhibition of FAK expression, its downstream signaling, and the invasive potential of the OS cells, resulting in decrease in metastatic lesions.

**Conclusion:**

The present study might lead not only to the clarification of a new molecular mechanism of invasion and metastasis of OS, but also to the development of a new therapeutic strategy of blocking IL-8 in OS.

## Background

Normal cells adjacent to tumors are believed to be under the influence of the tumor cells via direct contact. Indeed, it has been demonstrated by use of various malignant tumors that mesenchymal stromal cells surrounding the tumor are affected by the tumor to consequently aid tumor proliferation [1, 2]. The interaction is considered to occur primarily between the tumor cells and directly contacting cells [3]. However, if this interaction is mediated by a humoral factor that can disperse to a wide range, it might be remarkably advantageous for the environmental improvements in tumor expansion including distant metastasis. It is possible that the tumor cells that have successfully acquired such ability to utilize humoral factors spread selectively.

In the present study, we hypothesized that humoral factors might be involved in more efficient modification, by OS cells, of the microenvironment and/or even the condition of the distal metastatic destination favorably for the tumor. On the basis of this concept, we developed a co-culture model of the human OS cell line MG63 and human mesenchymal stem cells (hMSCs). We comprehensively analyzed changes in mRNA expression in both cell lines of independent culture and co-culture conditions by means of cDNA array. The results demonstrated that the co-culture induced high expression of IL-8 in both cell lines, and that IL-8 functioned as a ligand leading to the phosphorylation of focal adhesion kinase (FAK) and activation of motility of OS cells [4, 5]. We further found that the paracrine factor IL-8 formed a signaling loop between OS cells and hMSCs, leading to the tumor progression and metastatic spread. Understanding the molecular mechanisms that drive metastatic potential via communication by humoral factors between OS cells and hMSCs will be important for the identification of new targets for prevention of metastasis.

## Results

### Higher expression levels of IL-8 in MG63 than in hMSCs

**Fig. 1:**
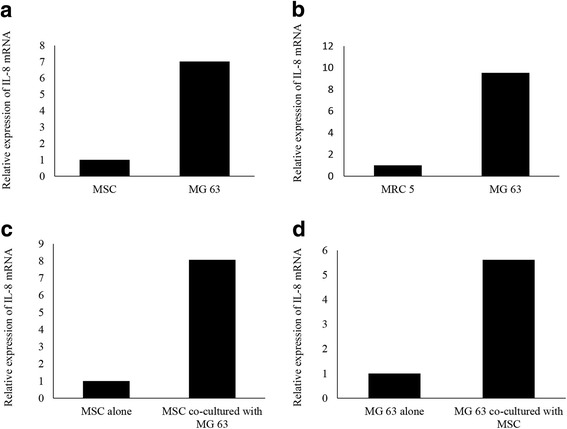
The expression of IL-8 in mono-cultured and co-cultured osteosarcoma and normal cells. **a** Relative expression of IL-8 mRNA in hMSC and MG63. **b** Relative expression of IL-8 mRNA in MRC 5 and MG63. **c** Changes in gene expression in hMSCs after the co-culture with MG63. **d** Changes in gene expression in MG63 after the co-culture with hMSCs

The genome-wide cDNA expression profiling using MG63 was carried out to identify mRNAs specifically expressed in this OS cell line. The array analysis showed that the expressions of 6542 mRNAs in OS cells were significantly changed (fold-change > 2.0) in comparison with that in hMSCs. Among the 6542 mRNAs, 2801 were up-regulated, whereas 3741 were down-regulated in MG63 cells compared to that in hMSCs. Regarding humoral factors, the expression of IL-8 was most up-regulated among the cytokines and growth factors. The IL-8 expression level of MSC was 7.02 times greater than MG63 monoculture (Fig. 1a), and the fibroblasts MRC5 were 9.54 greater (Fig. 1b).

### Up-regulation of IL-8 in hMSCs by co-culture with MG63

The cDNA array analysis demonstrated that the expressions of 6903 mRNAs were significantly changed (fold-change > 2.0) between hMSCs co-cultured with MG63 and hMSCs alone. We found that 3914 genes were significantly up-regulated, whereas 2989 genes were significantly down-regulated in hMSCs co-cultured with MG63 compared to that in hMSCs alone. Of the altered expression levels of humoral factors in the co-cultured condition, IL-8 was the most up-regulated among the cytokines and growth factors. The MSC IL-8 expression level increased 8.07 times after coculture compared to MSC alone (Fig. 1c).

### Up-regulation of IL-8 in MG63 by co-culture with hMSCs

The cDNA array analysis further demonstrated that the expressions of 7378 mRNAs were significantly changed (fold-change > 2.0) between MG63 co-cultured with hMSCs and MG63 alone. We found that 4117 genes were significantly up-regulated, whereas 3261 genes were significantly down-regulated in MG63 co-cultured with hMSCs compared to that in MG63 alone. MG63 IL-8 expression level after coculture increased by 5.61 times compared to MG63 alone (Fig. 1d).

### Up-regulation of IL-8 in hMSCs and MG63 by IL-8

**Fig. 2:**
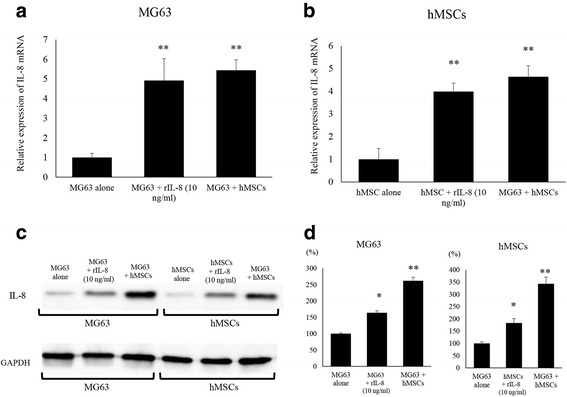
Changes in IL-8 expression in MG63 and hMSCs induced by the co-culture condition and rIL-8 administration. **a** Changes in IL-8 expression in MG63 were assessed by qRT-PCR. The co-culture and rIL-8 addition significantly increased the expression of mRNA of IL-8. (**) *p* < 0.01. **b** Changes in IL-8 expression in hMSCs were assessed by qRT-PCR. The co-culture and rIL-8 addition significantly increased the expression of mRNA for IL-8. (**) *p* < 0.01. **c** Changes in IL-8 protein expression of intra-cellular hMSCs and MG63 were assessed by western blot analysis. The co-culture and addition of rIL-8 increased the expression of IL-8 protein in MG63 and hMSCs. **d** The quantification of western blot analysis. Data represents represent the mean ± SD of three independent experiments. *p* < 0.05 was considered to indicate significance: (*) *p* < 0.05, (**) *p* < 0.01

An increase in IL-8 mRNA levels was observed after recombinant IL-8 (rIL-8) administration to MG63 and hMSC culture dishes. hMSCs treated with rIL-8 (399 ± 37%) and co-cultured with MG63 (464 ± 49%) showed significantly higher IL-8 mRNA levels than those in hMSCs alone (100%) (*p* < 0.01) (Fig. 2a). MG63 treated with rIL-8 (492 ± 84%) and co-cultured with MG63 (544 ± 53%) also showed significantly higher IL-8 mRNA levels than those of MG63 alone (*p* < 0.01) (Fig. 2b). We further performed immunoblot analysis to evaluate the protein levels of IL-8 in these cells (Fig. 2c). Western blot analysis showed that the expression levels of IL-8 in MG63 were significantly increased with rIL-8 administration (164 ± 6%) and MG63 co-cultured with hMSCs (262 ± 11%) compared to levels with MG63 alone. IL-8 expression levels were significantly increased after rIL-8 administration to hMSCs (183 ± 18%) and hMSCs co-cultured with MG63 (343 ± 27%) (*p* < 0.05) (Fig. 2d). These observations suggested that a stimulation loop of IL-8 expression might exist between MG63 and hMSCs.

### Inhibition of IL-8 expression by anti-IL-8 antibodies

**Fig. 3:**
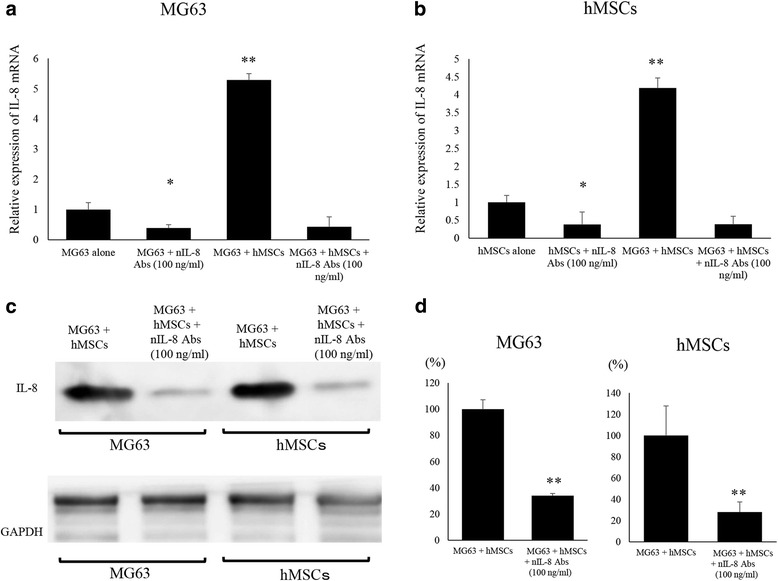
Effects of neutralizing anti-IL-8 Ab on IL-8 expression in mono-cultured and co-cultured MG63 and hMSCs. **a** Changes in IL-8 expression in MG63 were assessed by qRT-PCR. The addition of nIL-8 Ab to MG63 decreased the expression of IL-8 mRNA. **b** Changes in IL-8 expression in hMSCs were assessed by qRT-PCR. The addition of nIL-8 Ab to hMSCs decreased the expression of IL-8 mRNA. **c** Changes in IL-8 protein expression in hMSCs and MG63 were assessed by western blot analysis. The addition of nIL-8 Ab to MG63 and hMSCs decreased the expression of IL-8 protein in these cells. **d** The quantification of western blot analysis. Data represents represent the mean ± SD of three independent experiments. *p* < 0.05 was considered to indicate significance: (*) *p* < 0.05, (**) *p* < 0.01

Blockade of the IL-8 communication loop using anti-IL-8 mAb resulted in reduced IL-8 levels in both MG63 and hMSC in co-culture. Indeed, MG63 co-cultured with hMSCs and treated with anti-IL-8 Ab showed lower IL-8 mRNA levels (39 ± 2.8%) than untreated MG63 co-cultured with hMSCs (*p* < 0.05) (Fig. 3a). hMSCs co-cultured with MG63 and treated with anti-IL-8 Ab exhibited lower IL-8 mRNA levels (43 ± 33%) than hMSCs co-cultured with MG63 (*p* < 0.01) (Fig. 3b). Western blot analysis showed that IL-8 protein expression levels in co-cultured MG63 and hMSCs were dramatically decreased with anti-IL-8 Ab administration (*p* < 0.05) (Fig. 3c). IL-8 expression levels were significantly decreased after nIL-8 Abs administration to MG63 (34 ± 1.7%) and MG63 co-cultured with hMSCs and treated with anti-IL-8 Ab compared to levels with MG63 alone. IL-8 expression levels were significantly decreased after nIL-8 Abs administration to MG63 (28 ± 9.7%) and MG63 co-cultured with hMSCs and treated with anti-IL-8 Ab compared to levels with hMSCs alone (*p* < 0.01) (Fig. 3d).

### Effects of co-culture and IL-8 on MG63 cell growth

**Fig. 4:**
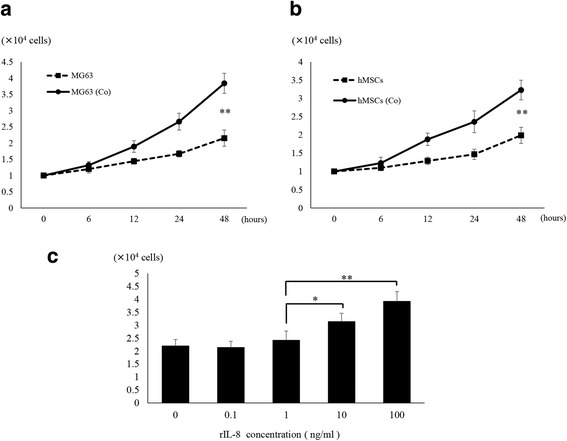
Changes in cell growth of MG63 cells and hMSCs induced by co-culture conditions and rIL-8 addition. **a** Co-culture with hMSCs significantly increased the cell growth of MG63 (MG63(Co)). (*) *p* < 0.05, (**) *p* < 0.01. **b** Co-culture with MG63 significantly increased the cell growth of hMSCs (hMSCs(Co)). (*) *p* < 0.05, (**) *p* < 0.01. **c** rIL-8 was administered to mono-cultured MG63. There was a significant increase in cell growth with rIL-8 at 10 ng/ml. (*) *p* < 0.05, (**) *p* < 0.01

We next examined the effects of the co-cultured condition and IL-8 on the proliferation of MG63 OS cells. The cell growth at 48 h of co-cultured MG63 (3.99 ± 0.37 × 10^5^ cells) was significantly increased in comparison to that with MG63 alone (2.15 ± 0.25 × 10^5^ cells) (*p* < 0.01) (Fig. 4a). The cell growth at 48 h of co-cultured hMSCs (3.23 ± 0.27 × 10^5^ cells) was also significantly increased compared to that with hMSCs alone (1.99 ± 0.22 × 10^5^ cells) (*p* < 0.01) (Fig. 4b). Furthermore, the cell growth of MG63 was significantly increased by administration of rIL-8 at 10 ng/ml (3.14 ± 0.32 × 10^5^ cells) compared with 1 ng/ml (2.42 ± 0.35 × 10^5^ cells) as determined by cell counting 48 h after the administration (*p* < 0.05) (Fig. 4c).

### Intensification of cell motility of MG63 by co-culture and IL-8

**Fig. 5:**
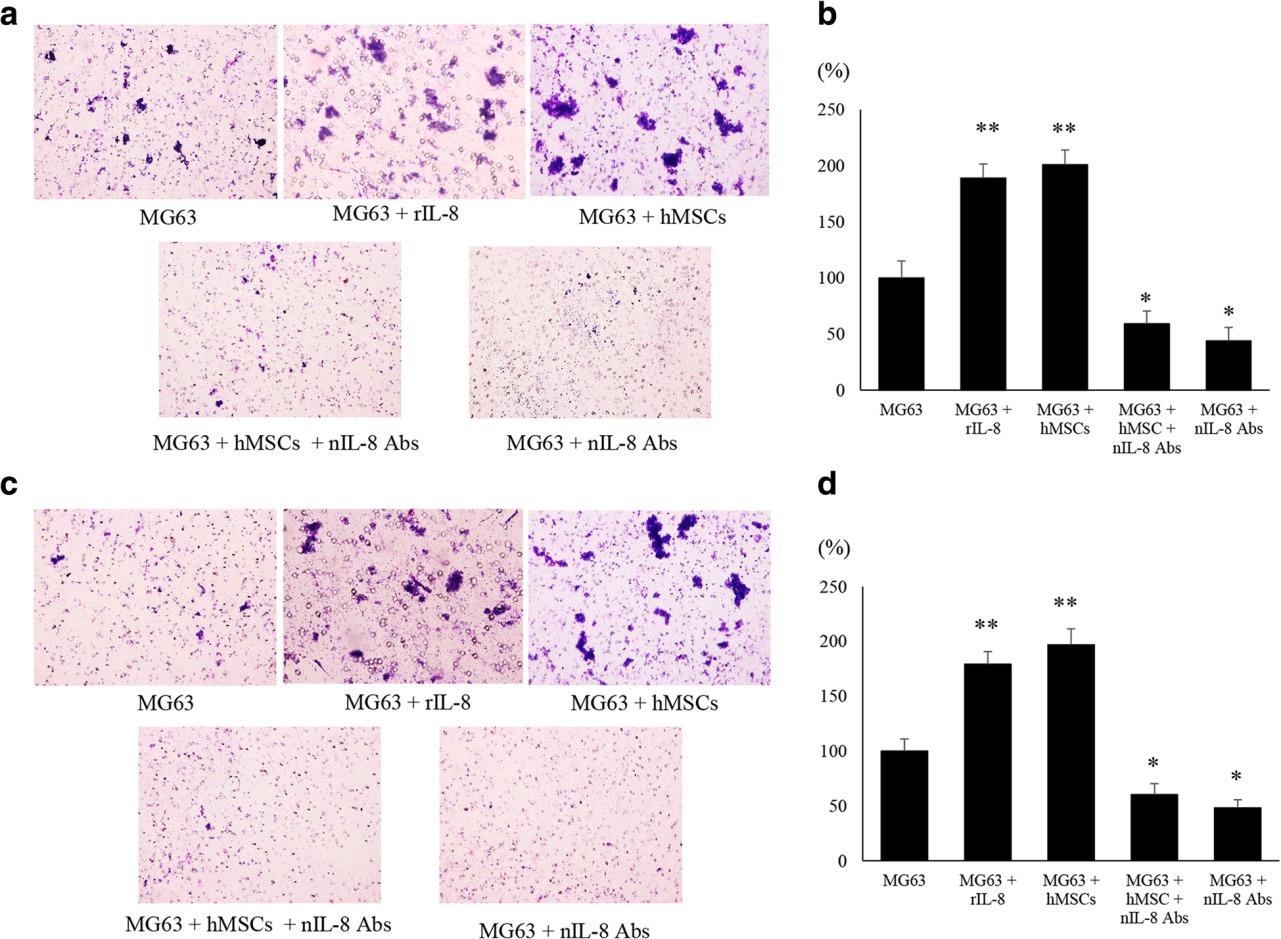
Effects of co-culture condition and administration of rIL-8 or anti-IL-8 Ab on motility of MG63. **a** The cell motility of MG63 was assessed in each group at 48 h after the challenge with or without rIL-8 and neutralizing anti-IL-8 antibodies (nIL-8 Ab). **b** The amount of MG63 cells that crossed the membrane was measured. A significant decrease in motility was found in the group given nIL-8 Ab. (*) *p* < 0.05, (**) *p* < 0.01. **c** The cell invasion in MG63 was assessed in each group after 48 h. **d** The amount of the cells of which the membrane with Matrigel was crossed by MG63 was measured. Decreased migration ability was found in the group administered anti-IL-8 Ab. (*) *p* < 0.05, (**) *p* < 0.01

To confirm the influence of IL-8 on cell motility and invasion, we performed a transwell motility assay using co-cultured MG63 and hMSCs with or without rIL-8 and anti-IL-8 Ab (Fig. 5a). MG63 cells after pulsing with rIL-8 (189.1 ± 12.5%) and MG63 after co-culturing with hMSCs (201 ± 13.3%) showed statistically increased motility capacity compared to MG63 alone (100%) (*p* < 0.01). MG63 co-cultured with hMSCs and with anti-IL-8 Ab (59.2 ± 11.5%) presented a more impaired motility capacity than those of MG63 co-cultured with hMSCs (201 ± 13.2%) in comparison with MG63 alone (100%) (*p* < 0.05) (Fig. 5b). The migration assay also demonstrated that MG63 co-cultured with hMSCs (Fig. 5c). MG63 cells after pulsing with rIL-8 (179.3 ± 11.6%) and MG63 after co-culturing with hMSCs (196.9 ± 14.6%) showed statistically increased migration capacity compared to MG63 alone (100%) (*p* < 0.01). Anti-IL-8 Ab (60.3 ± 9.9%) exhibited significantly lower motility than that of MG63 co-cultured with hMSCs (196.9 ± 14.6%) in comparison with MG63 alone (100%) (*p* < 0.05) (Fig. 5d).

### Changes in expression of FAK and its downstream factors

**Fig. 6:**
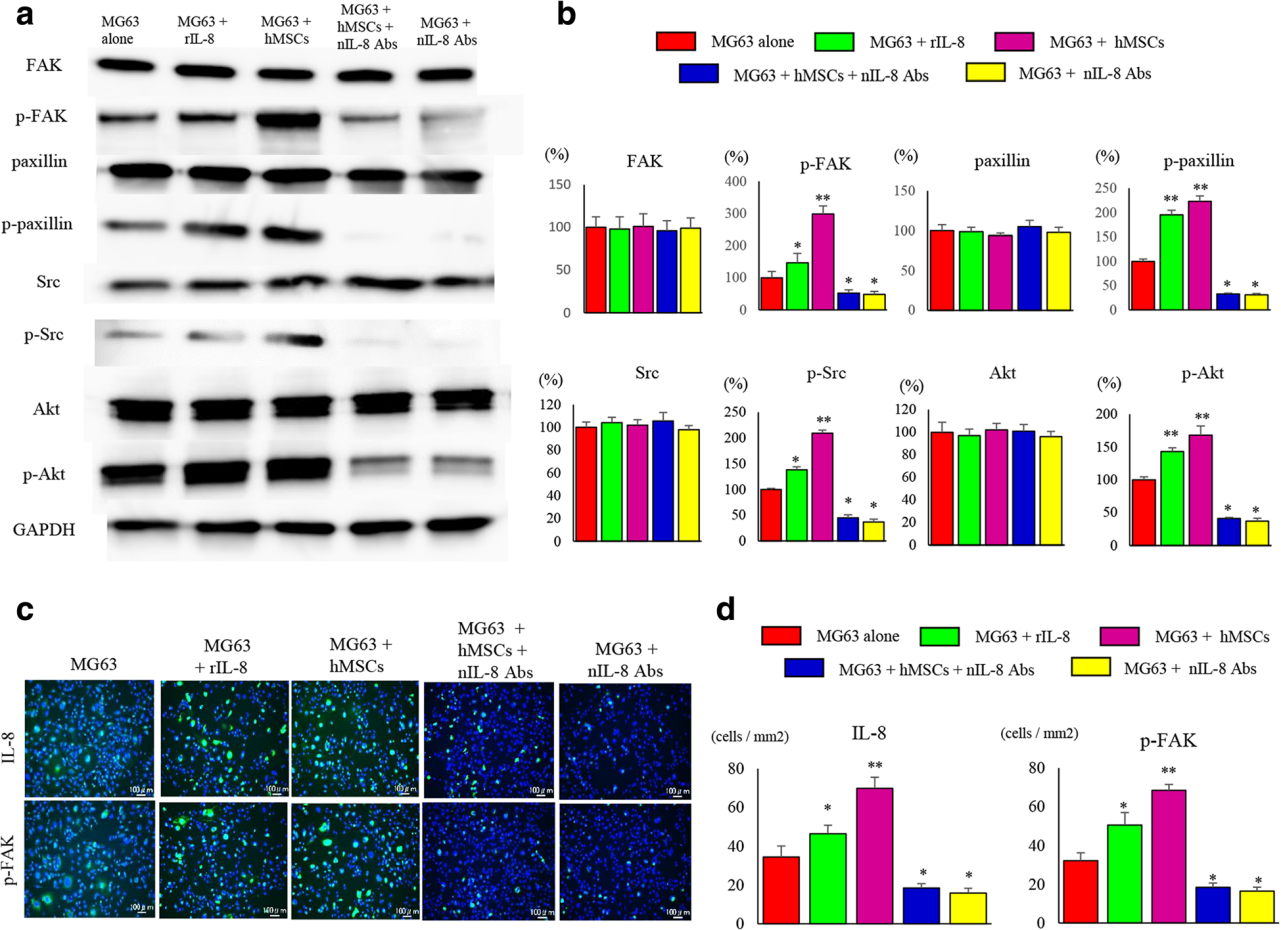
Changes in expression of FAK and its downstream factors related to invasive potential. **a** Changes in phosphorylation and the expression of protein factors relating to invasive potential were analyzed. Decreased phosphorylation of FAK, paxillin, Src, and Akt in MG63 cells was noted in the group administered nIL-8 Ab. **b** The quantification of western blot analysis. Data represents represent the mean ± SD of three independent experiments. *p* < 0.05 was considered to indicate significance: (*) *p* < 0.05, (**) *p* < 0.01. **c** Immunofluorescence staining of cultured MG63 cells showed decreased phosphorylation of FAK and paxillin in the group administered nIL-8 Ab. **d** The number of IL-8 and p-FAK positive cells per unit area. Data represents represent the mean ± SD of three independent experiments. *p* < 0.05 was considered to indicate significance: (*) *p* < 0.05, (**) *p* < 0.01

Since cell motility was remarkably enhanced in co-cultured MG63 with hMSCs, and FAK is known to be a key regulatory molecule for cell motility, immunoblot analyses were carried out to investigate the expression of FAK and its downstream factors (Fig. 6a). The protein expression levels of the phosphorylated form of FAK (147 ± 29.4%), paxillin (195 ± 9.6%), Src (138.2 ± 3.1%), and Akt (143 ± 5.9%) in MG63 were elevated by the presence of rIL-8. The protein expression levels of the phosphorylated form of FAK (298 ± 25.3%), paxillin (223 ± 11.2%), Src (209.6 ± 5.7%), and Akt (168 ± 14.1%) in MG63 were elevated by the co-culture with hMSCs. However, the expression levels of the phosphorylated form of FAK (48.5 ± 9.6%), paxillin (31.4 ± 2.9%), Src (37 ± 5.3%), and Akt (38.6 ± 4.3%) in MG63 were dramatically decreased with anti-IL-8 Ab administration compared with MG63 alone cells. The expression levels of the phosphorylated form of FAK (52.8 ± 10.4%), paxillin (33.2 ± 1.5%), Src (45.6 ± 5.7%), and Akt (41 ± 1.8%) were dramatically decreased in co-cultured cells with anti-IL-8 Ab administration compared with MG63 alone (*p* < 0.05) (Fig. 6b). Immunofluorescence analysis further revealed that the phosphorylation levels of FAK and paxillin decreased in anti-IL-8 Ab-treated MG63 cells compared to those in mono- or co-cultured cells. These data indicated that the phosphorylation levels of factors related to cell motility were reduced in the anti-IL-8 Ab-treated cells (Fig. 6c). The number of cells positive for IL-8 expression was significantly increased in administered with rIL-8 (46.3 ± 4.4 cells/mm^2^) or MG63 co-cultured with hMSCs cells (69.8 ± 5.7 cells/mm^2^) compared to MG 63 alone (34.5 ± 5.5 cells/mm^2^). The number of cells positive for IL-8 expression was significantly decreased administered nIL-8 Abs with MG63 (15.8 ± 2.4 cells/mm^2^) or MG63 co-cultured with hMSCs cells administered nIL-8 Abs (18.5 ± 2.5 cells/mm^2^) compared to MG 63 alone (*p* < 0.05). The number of cells positive for p-FAK expression was significantly increased administered with rIL-8 (50.5 ± 6.5 cells/mm^2^) or MG63 co-cultured with hMSCs cells (68.5 ± 3.1 cells/mm^2^) compared to MG 63 alone (32.2 ± 3.9 cells/mm^2^). The number of cells positive for IL-8 expression was significantly decreased administered nIL-8 Abs with MG63 (16.5 ± 2.1 cells/mm^2^) or MG63 co-cultured with hMSCs cells administered nIL-8 Abs (18.5 ± 2.2 cells/mm^2^) compared to MG 63 alone (*p* < 0.05). (Fig. 6d).

### Inhibition of tumor metastasis in a nude mice xenograft model by IL-8 suppression

**Fig. 7:**
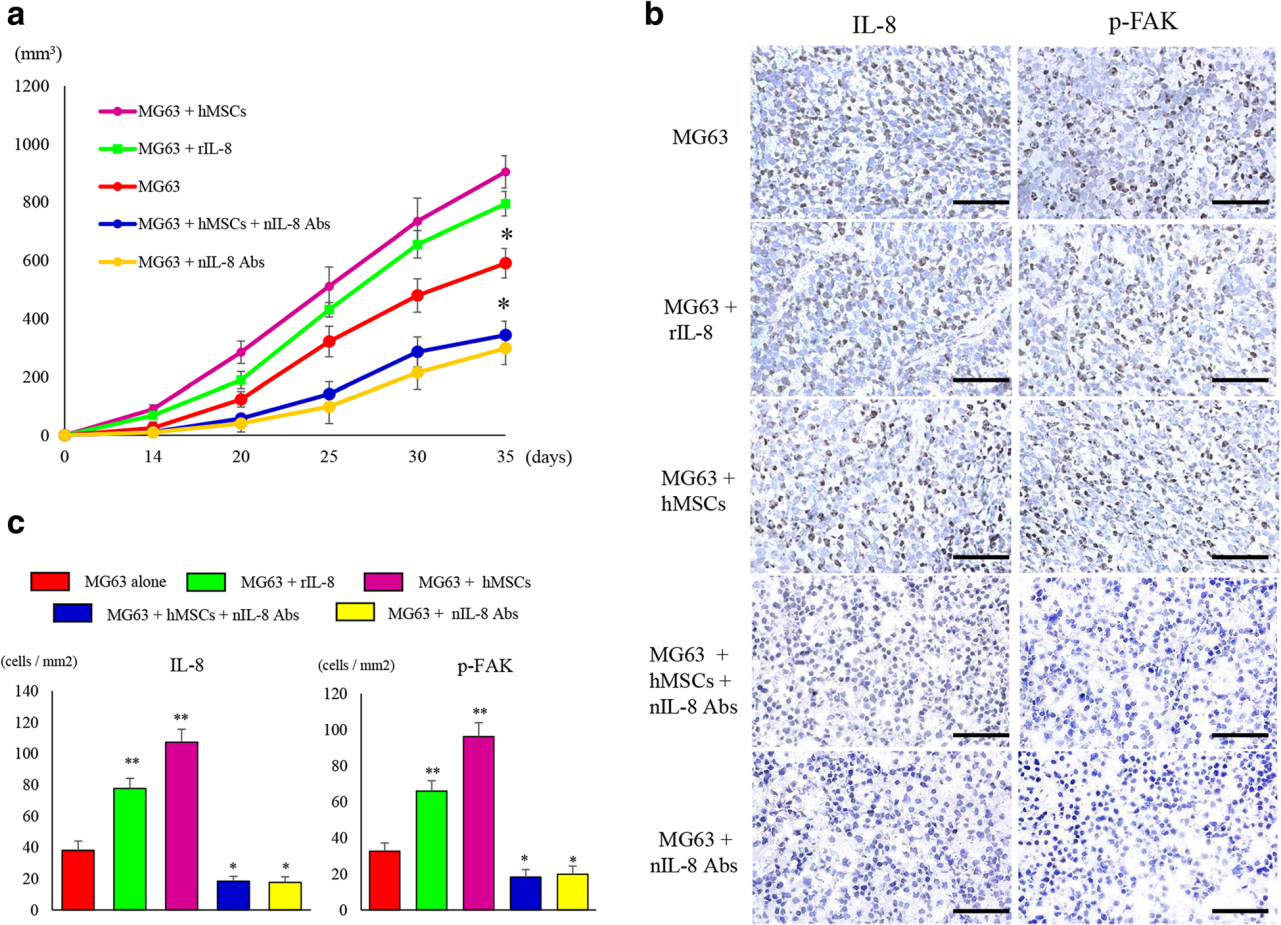
Changes in the lung nodules and the expression of IL-8 and phosphorylated FAK in pulmonary metastatic lesions. **a** The group given nIL-8 Ab showed a significant suppression of the size of the pulmonary metastatic lesion. (*) *p* < 0.05. **b** Immunostaining of the tissues collected from the pulmonary metastatic lesion. Decreased expression of IL-8 and phosphorylation of FAK was observed in the group administered nIL-8 Ab. Original magnification, × 400; Scale bars: 50 μm. **c** The number of IL-8 and p-FAK positive cells per unit area. Data represents represent the mean ± SD of three independent experiments. *p* < 0.05 was considered to indicate significance: (*) *p* < 0.05, (**) *p* < 0.01

We next investigated the efficacy of IL-8 silencing against OS tumor metastasis in vivo (Fig. 7a). The suppression of IL-8 by anti-IL-8 Ab in MG63 cells co-cultured with hMSCs resulted in a significant decrease in the growth of metastatic tumors in nude mice. MG63 cells after pulsing with rIL-8 for 48 h (794.6 ± 42.1 mm^3^) and MG63 after co-culturing with hMSCs for 48 h (904.4 ± 55.4 mm^3^) showed statistically larger lung tumors in mice compared to MG63 alone (590.9 ± 50.8 mm^3^). On the other hand, MG63 cells after administration of anti-IL-8 Ab (343.6 ± 47.8 mm^3^) and co-cultured MG63 treated with anti-IL-8 Ab (298.4 ± 55.9 mm^3^) showed statistically smaller lung tumors in mice compared to MG63 alone. Immunohistochemistry analysis using resected lung tumors demonstrated that the expression of IL-8 and p-FAK was reduced in the anti-IL-8 Ab-administered tumor tissues (Fig. 7b). The number of cells positive for IL-8 expression was significantly increased in mice administered with rIL-8 (77.6 ± 6.5 cells/mm^2^) or MG63 co-cultured with hMSCs cells (107.4 ± 8.4 cells/mm^2^) compared to MG 63 alone (38.2 ± 5.8 cells/mm^2^).

The number of cells positive for IL-8 expression was significantly decreased in mice administered nIL-8 Abs with MG63 (17.7 ± 3.6 cells/mm2) or MG63 co-cultured with hMSCs cells administered nIL-8 Abs (18.5 ± 3.1 cells/mm2) compared to MG 63 alone (*p* < 0.01). The number of cells positive for p-FAK expression was significantly increased in mice administered with rIL-8 (66 ± 5.6 cells/mm^2^) or MG63 co-cultured with hMSCs cells (96.1 ± 7.8 cells/mm^2^) compared to MG 63 alone (32.7 ± 4.4 cells/mm^2^). The number of cells positive for p-FAK expression was significantly decreased in mice administered nIL-8 Abs with MG63 (19.8 ± 4.6 cells/mm^2^) or MG63 co-cultured with hMSCs cells administered nIL-8 Abs (18.3 ± 4.1 cells/mm^2^) compared to MG 63 alone (*p* < 0.05). (Fig. 7c).

## Discussion

Since the pathology of malignant tumors involves various normal cells in the vicinity and microenvironmental factors [6, 7], it is insufficient to analyze only tumor cells to understand the mechanisms of tumor progression and metastasis. When considering how tumor cells interact with their neighboring cells in vivo, particularly in the establishment of metastasis, it is important to determine the significance of signal cross-talk in the condition in which the parties are apart mutually. The present study, using the co-culture system, aimed to identify humoral factors that play a role in interactions between OS and its surrounding microenvironment, leading to elucidation of the molecular mechanisms of interactions with the vicinity cells, as well as the biological roles in distant metastasis and others.

It has been reported that normal cells can be influenced by humoral factors from tumors, which lead to the normal cells acquiring a function to support the tumor cells [8, 9]. Thus, it is likely that genes changing their expression levels in the co-culture condition might be the key players in the cross-talk mechanism between tumor and normal cells. In the present study, our data revealed that both MG63 cells and hMSCs released IL-8 and influenced each other via an IL-8 signaling loop, which caused possible changes in the ecology of tumors. We believe that the results would contribute towards elucidation of the molecular mechanisms of tumor progression that occur in the microenvironment.

The results of cDNA array analyses demonstrated that IL-8 mRNA expression was significantly increased in MG63 co-cultured with hMSCs. Since elevated IL-8 within the microenvironment of many cancers is known to enhance tumor progression [10], we further characterized its role in response to IL-8 in hMSCs. Our data suggested that up-regulation of IL-8 in co-cultured hMSCs, as well as in co-cultured MG63 cells, may contribute to the stimulation of some malignant potentials of MG63.

We also analyzed the paracrine action of IL-8 between MG63 and hMSCs in the co-cultured condition. When rIL-8 was added to mono-cultured MG63 and hMSCs separately, the intracellular protein production of IL-8 in MG63 and hMSCs was up-regulated by the administration of rIL-8, as in the co-culture condition, compared with mono-cultured MG63 and hMSCs. The results suggested that both MG63 cells and hMSCs might utilize IL-8 for the production of IL-8 itself in a signaling loop in a paracrine manner in the co-culture condition.

To confirm whether elevated IL-8 levels would mediate the enhancement of the cancer activities in MG63 co-cultured with hMSCs, we added a neutralizing antibody against IL-8 (nIL-8 Ab) and observed the changes in biological characteristics of hMSCs co-cultured with MG63. Interestingly, IL-8 mRNA and protein levels in co-cultured MG63 were remarkably down-regulated by the administration of anti-IL-8 Ab. The results suggested the possibility of not only a paracrine but also an autocrine system by which MG63 and hMSCs produce IL-8 by the stimulation of IL-8 itself. Based on the results of using neutralizing antibodies, an autocrine mechanism is thought to be involved in both OS and MSC. MG63 normally releases more IL-8 than MSC, and it is thought that it utilized both autocrine and paracrine mechanisms for its own growth. In contrast to this, when IL-8 stimulus is added to MSC, its own IL-8 production volume also increases. The addition of nIL-8 Ab significantly inhibited the motility of MG63 co-cultured with hMSCs. Our data was consistent with that of previous reports showing that IL-8 is necessary for the mobility of tumor cells [11, 12]. On the other hand, rIL-8 significantly increased the ability of invasion in MG63 in accordance with the previous studies regarding metastasis in gastric cancer [13], colon cancer [14], and head and neck squamous cell carcinoma [15]. Invasive capability analysis was conducted via the number of cells that passed through the Matrigel, which has been evaluated as being related to metastatic capability in other studies as well [16, 17]. Since IL-8 and its downstream protein tyrosine kinases are involved in invasion and metastasis, which could be suppressed by targeting IL-8 [18], our results in MG63 suggest the same possibility for a therapeutic strategy for OS.

The factors controlling invasive potential of malignant tumor cells include FAK. We verified whether or not IL-8 was responsible for phosphorylation of FAK, and found that FAK and its downstream molecules were phosphorylated by rIL-8 as well as in the co-cultured condition in which invasive potential of MG63 increased, and that those were inhibited by the neutralizing anti-IL-8 antibody administration. In addition, Akt downstream of FAK underwent phosphorylation mediated by IL-8, consistent with the results of cell proliferation. Our data suggested that the potential of invasion and proliferation of OS cells might increase through IL-8-induced activation of FAK and Akt signaling.

We further showed a reduction in the formation of lung metastasis in mice xenograft models co-injected with co-cultured MG63 and nIL-8 Ab. It has been demonstrated that the blockade of IL-8 resulted in significant anti-tumor effects in a xenograft model of cervical cancer [15]. Our study also showed an important tumor suppressor role for MG63, whose partner’s IL-8 abrogate via IL-8 neutralization, indicating that anti-IL-8 Ab could efficiently attenuate the in vivo metastasis of MG63 cells in collusion with hMSCs. Moreover, we confirmed significantly higher IL-8 release and FAK phosphorylation in metastatic lesions in the lung in vivo as well as in vitro. The present study demonstrated that IL-8 might be an important humoral factor for the metastatic activity of OS cells.

IL-8 is one of the most essential factors for many important tumorigenic phenotypes including proliferation, invasion, and migration. Increased expression of IL-8 is correlated with poor prognosis [19, 20], and a high level of IL-8 sustains cancer to be resistant to chemotherapy [21]. We ascertained that IL-8 plays an extremely important role as a humoral factor in the progression of osteosarcoma. That is, therapeutic strategies targeting IL-8 may enable establishment of a different therapeutic approach for cases resistant to current anti-cancer drugs. To the best of our knowledge, our study is the first report to show that IL-8 could be a therapeutic target of human OS using a co-culture system with OS cells and hMSCs. Although our notions will require further investigation, the cytokines induced by OS may coordinate with cells in the vicinity and contribute to potentiating the invasion and angiogenesis required in the tumor microenvironment. Taken altogether, the present study provides evidence of cross-talk by IL-8 between hMSCs and OS cells.

## Methods

### Cell lines

The human osteosarcoma cell line MG63 was obtained from RIKEN Cell Bank (Tsukuba, Japan). hMSCs were purchased from TaKaRa Biotechnology (Otsu, Japan). Each line was authenticated as to genotype and phenotype by the source company. MG63 cells were cultured in Dulbecco’s modified Eagle medium (DMEM) - high glucose (Invitrogen, NY, USA) with 10% FBS and 1% penicillin and streptomycin. hMSCs were cultured with Mesenchymal Stem Cell Basal Medium, Chemically Defined (MSCBM-CD) with MSCGM-CD SingleQuats (TaKaRa Bio). The cells were maintained at 37 °C in an incubator supplied with 5% CO_2_ and passaged every 2 to 3 days.

### Co-cultured condition

hMSCs and MG63 cells were seeded at 1 × 10^5^ cells/well individually during polymerization of the collagen type I lattice and cultured on opposite sides of a 1-μm pore, six-well cell culture insert (Becton Dickinson, Sparks, MD, USA). Cells were incubated for 48 h at 37 °C and 5% CO_2_, and total RNA was isolated from two inserts for each cell type. All experiments were performed in duplicate.

### RNA isolation

mRNAs were prepared from the triplicated cell cultures using an RNeasy kit (Qiagen, Valencia, CA, USA) according to the manufacturer’s instructions. The RNA quality was ensured, before labeling, using an RNA 6000 Nano kit and a Bioanalyzer 2100 (Agilent, Santa Clara, CA, USA).

### Analysis of mRNA expression by cDNA arrays

GeneChip Genome HG U133 Plus 2.0 Array (Affymetrix) was used for mRNA expression profiling in MG63 and hMSCs. Biotinylated cRNA was synthesized from total RNA using the 3’ IVT Express Kit (Affymetrix) according to the manufacturer’s protocols. In brief, double stranded cDNA was generated by reverse transcription from 1 ng of total RNA using an oligo(dT) primer bearing a T7 promotor. The double-strand cDNA was used as a template for in vitro transcription to generate biotin-labeled cRNA. After fragmentation, 12.5 μg of cRNA was hybridized to the GeneChip array for 16 h. The arrays were washed and stained using GeneChip Fluidics Station 450 (Affymetrix) and then scanned with the GeneChip Scanner 3000 (Affymetrix). The entire experiment was performed twice. Array hybridization, washing, and scanning of the slides were carried out according to the manufacturer’s protocols. The microarray numerical values were analyzed using the GeneSpring GX 11.0 software, according to the RAM16 Algorithm: quantile normalization, filter by flags (detected), and filter by expression on the normalized data (20.0–100.0th percentile). Analysis of variance was used to determine those probe sets that were significantly different between the two groups. The gene list was filtered with a fold-change cutoff of 2, resulting in the output of a list with genes that had significant differential expression at 2-fold or greater differences.

### Recombinant IL-8 administration

rIL-8 (R&D Systems; Minneapolis, MN, USA) (10 ng/ml) was administered to the culture medium. After 48 h of incubation following the administration, the cells were harvested and processed for further analysis. The experiment was repeated three times.

### Neutralization of IL-8 function using anti-IL-8 antibody

Neutralizing monoclonal antibody targeting IL-8 (anti-IL-8 Ab) was purchased from R&D Systems. The cell lines were harvested 48 h after the administration of IL-8 and anti-IL-8 Ab (100 ng/ml), then subjected to various analyses. The experiment was repeated three times.

### Cell proliferation assay

The cells were plated in 6-well plates (1 × 10^5^ cells per well), and were treated with or without rIL-8 and neutralizing anti-IL-8 Ab. The concentrations of rIL-8 added to MG63 cells were 0, 0.1, 1, 10, 100 ng/ml. After 48 h of cultivation, the cells were counted using a TC10 Automated Cell Counter (Bio-Rad).

### Quantitative real time PCR

Total RNA was extracted from prepared cultured cells with TRIzol reagent (Invitrogen) and cDNA was synthesized according to the manufacturer’s protocol (Roche). Quantitative real-time PCR (qRT-PCR) was performed using a Light Cycler 480 Probe Master System (Roche), and PCR-specific amplification was conducted using the LightCycler® Nano (Roche). The relative expression of IL-8 and glyceraldehyde-3-phosphate dehydrogenase (GAPDH) was calculated using the 2-(ΔΔCt) method method. The primers and probe kits of IL-8 and GAPDH were obtained from Applied Biosystems (Nagoya, Japan).

### Cell motility and migration assays

For the motility assays, 5 × 10^4^ cells were suspended in the migration medium (medium without FBS), and were plated in the top chamber without the Matrigel-coated membrane (24-well insert; 8-μm pore size; BD Biosciences). The lower compartment was filled with 600 μl of a medium containing 30% FBS as a chemo-attractant. Following incubation for 16 h at 37 °C and 5% CO_2_ in a humidified incubator, the cells on the lower surface of the filter were fixed in 4% formaldehyde for 20 min, then stained with Giemsa stain for 10 min at room temperature, and examined by light microscopy.

### Western blot

Whole cell lysates were prepared from MG63 and hMSCs, and cellular protein (15 μg) was resolved on a precast 10% Tris–HCl Criterion 10-well gel (Bio-Rad) at 200 V (300 mAmp) for 30 min. Antibodies against IL-8 proteins (R&D Systems) were obtained from Abcam (Cambridge, UK). Antibodies Rabbit anti - Human FAK (#3285), paxillin (# 2542), Src (#2108), Akt (#4691), phosphorylated (p)-FAK (#3283S), p-paxillin (#2541), p-Src (#5473), p-Akt (#4060), GAPDH (#5174), PARP (#9542), and cleaved-PARP (#5625) were obtained from Cell Signaling Technology (Tokyo, Japan). Immunocomplexes were visualized with horseradish peroxidase-conjugated anti-rabbit immunoglobulin G antibodies (GE Healthcare, Tokyo, Japan), and blots were developed using an ECL Plus system (GE Healthcare) with a ChemiDoc camera (ImageQuant LAS 4000mini; GE Healthcare). Quantification of western blot signals was performed by densitometry with ImageQuant TL software (GE Healthcare). All primary antibodies were used at a 1:1000 dilution. Peroxidase-conjugated anti-Rabbit IgG secondary antibodies (GE Healthcare) were used at a 1:2000 dilution. Three independent experiments were performed for each analysis, and the same experimental conditions were used for all gels.

### Immunofluorescence analysis

Immunohistochemistry was used to measure the levels of p-FAK and p-paxillin in the cells. After PBS washing, rehydrated culture dishes were incubated with primary antibodies diluted at 1:200 in Ab Diluent (Dako ChemMate; Dako, Japan) overnight at room temperature. For staining with Alexa Fluor 488 anti-rabbit IgG (Invitrogen, Carlsbad, CA, USA), secondary antibodies were diluted at 1:300 in Ab Diluent and added for 60 min at room temperature in the dark. Digital images were taken on a BIOREVO microscope equipped with a confocal microscopy system (BZ-9000, Keyence, Japan).

### In vivo tumor-bearing nude mouse model

The experimental metastasis model was established by injection of 1 × 10^6^ cells suspended in 100 μl of normal saline into the tail veins of nude mice. Five groups were generated: (1) untreated MG63 cells (MG63) (*n* = 5); (2) MG63 cells treated with rIL-8 (MG63 + rIL-8) (n = 5); (3) MG63 cells co-cultured with hMSCs (MG63 + hMSCs) (n = 5); (4) MG63 cells co-cultured with hMSCs and treated by anti-IL-8 Ab (MG63 + MSCs + anti-IL-8 Ab) (n = 5); and (5) MG63 cells treated with anti-IL-8 Ab (MG63 + anti-IL-8 Ab) (n = 5). All mice were fed in standard conditions with weight monitoring, and sacrificed 6 weeks after the cell inoculation. All mice used in this study were anesthetized with ketamine/xylazine or isoflurane/oxygen for experiments. Tumor volumes were measured using a micro-CT apparatus (R_mCT) which allows us to obtain high-resolution CT images in small living animals. The tumor volume of the lung nodule was estimated using the formula: (π × long axis × short axis × short axis)/6.

### Statistical analysis

Statistical analysis was carried out using SPSS 22.0 software (SPSS Japan Inc., Tokyo, Japan). A two-tailed Student’s t-test was used for the analysis of continuous variables. We determined the differences among more than three groups using a non-repeated measures analysis of variance (ANOVA) and Scheffe test. Results were expressed as the mean ± standard deviation, and *p* < 0.05 was considered as statistically significant.

## Abbreviations

ANOVA: Analysis of variance
FACS: Fluorescence activated cell sorting
hMSCs: Human mesenchymal stem cells
NC: Negative control
OS: Osteosarcoma
PI: Propidium iodide
qRT-PCR: Quantificational real-time polymerase chain reaction

## References

[1] De Wever O, Mareel M (2003). Role of tissue stroma in cancer cell invasion. J Pathol.

[2] Ruiter D, Bogenrieder T, Elder D, Herlyn M (2002). Melanoma-stroma interactions: structural and functional aspects. Lancet Oncol.

[3] Gupta GP, Massagué J (2006). Cancer metastasis: building a framework. Cell.

[4] Liu S, Ginestier C, Ou SJ, Clouthier SG, Patel SH, Monville F (2011). Breast cancer stem cells are regulated by mesenchymal stem cells through cytokine networks. Cancer Res.

[5] Bohrer LR, Schwertfeger KL (2012). Macrophages promote fibroblast growth factor receptor-driven tumor cell migration and invasion in a CXCR2-dependent manner. Mol Cancer Res.

[6] Han S, Xu W, Wang Z, Qi X, Wang Y, Ni Y (2016). Crosstalk between the HIF-1 and toll-like receptor/nuclear factor-κB pathways in the oral squamous cell carcinoma microenvironment. Oncotarget.

[7] Hartmann S, Bhola NE, Grandis JR (2016). HGF/met signaling in head and neck Cancer: impact on the tumor microenvironment. Clin Cancer Res.

[8] Zambirinis CP, Levie E, Nguy S, Avanzi A, Barilla R, Xu Y (2015). TLR9 ligation in pancreatic stellate cells promotes tumorigenesis. J Exp Med.

[9] Patel SA, Gooderham NJ (2015). IL6 mediates immune and colorectal Cancer cell cross-talk via miR-21 and miR-29b. Mol Cancer Res.

[10] Campbell LM, Maxwell PJ, Waugh D (2013). Rationale and means to target pro-inflammatory Interleukin-8 (CXCL8) signaling in Cancer. Pharmaceuticals (Basel).

[11] Kitadai Y, Takahashi Y, Haruma K, Naka K, Sumii K, Yokozaki H (1999). Transfection of interleukin-8 increases angiogenesis and tumorigenesis of human gastric carcinoma cells in nude mice. Br J Cancer.

[12] Strieter RM, Polverini PJ, Arenberg DA, Walz A, Opdenakker G, Van Damme J (1995). Role of C-X-C chemokines as regulators of angiogenesis in lung cancer. J Leukoc Biol.

[13] Kuai WX, Wang Q, Yang XZ, Zhao Y, Yu R, Tang XJ (2012). Interleukin-8 associates with adhesion, migration, invasion and chemosensitivity of human gastric cancer cells. World J Gastroenterol.

[14] Ning Y, Manegold PC, Hong YK, Zhang W, Pohl A, Lurje G (2011). Interleukin-8 is associated with proliferation, migration, angiogenesis and chemosensitivity in vitro and in vivo in colon cancer cell line models. Int J Cancer.

[15] Wu S, Shang H, Cui L, Zhang Z, Zhang Y, Li Y (2013). Targeted blockade of interleukin-8 abrogates its promotion of cervical cancer growth and metastasis. Mol Cell Biochem.

[16] Silvera D, Arju R, Darvishian F, Levine PH, Zolfaghari L, Goldberg J (2009). Essential role for eIF4GI overexpression in the pathogenesis of inflammatory breast cancer. Nat Cell Biol.

[17] Kajiro M, Hirota R, Nakajima Y, Kawanowa K, So-ma K, Ito I (2009). The ubiquitin ligase CHIP acts as an upstream regulator of oncogenic pathways. Nat Cell Biol.

[18] Yu Z, Willmarth NE, Zhou J, Katiyar S, Wang M, Liu Y (2010). microRNA 17/20 inhibits cellular invasion and tumor metastasis in breast cancer by heterotypic signaling. Proc Natl Acad Sci U S A.

[19] Reis ST, Leite KR, Piovesan LF, Pontes-Junior J, Viana NI, Abe DK (2012). Increased expression of MMP-9 and IL-8 are correlated with poor prognosis of bladder Cancer. BMC Urol.

[20] Yoon JY, Lafarge S, Dawe D, Lakhi S, Kumar R, Morales C (2012). Association of interleukin-6 and interleukin-8 with poor prognosis in elderly patients with chronic lymphocytic leukemia. Leuk Lymphoma.

[21] Gyanchandani R, Sano D, Ortega Alves MV, Klein JD, Knapick BA, Oh S (2013). Interleukin-8 as a modulator of response to bevacizumab in preclinical models of head and neck squamous cell carcinoma. Oral Oncol.

